# Supplementary material for: Controversies and Clinical Applications of Non-Invasive Transspinal Magnetic Stimulation: A Critical Review and Exploratory Trial in Hereditary Spastic Paraplegia
Source: J Clin Med. 2022 Aug 14;11(16):4748. doi: 10.3390/jcm11164748 (PMC9409717; doi:10.3390/jcm11164748)
Supplement: Supplementary file 1 [file jcm-11-04748-s001.zip › jcm-1804851-supplementary.pdf]

Supplementary Table S1. Relevant TsMS exploratory human studies.

Study	Population	Stimulation	Side effects	Relevant findings and conclusions
**Barker et al 1987 [3]**	15 multiple sclerosis patient, 27 healthy controls, 5 motor neuron disease	Circular coil single pulse, over cervical column	Minor discomfort	It was not possible to stimulate descending tracts in the spinal cord but roots or plexus were reliably stimulated. Central conduction times could be assessed comparing M1 and peripheral stimulation
**Ugawa et al 1989 [14]**	10 healthy controls	9 cm circular coil at various levels C2 to S1, compared to TES	None	No inferior limbs activity from cervical stimulation Fixed EMG latency when moving coil compatible with Spinal root activation. Overall similar latencies to TES. Maximum root activation when current flows outwards at the foramina
**Similowski et al 1989 [21]**	6 healthy controls	9 cm circular coil at C7 single pulses	None	Reliable diaphragmatic CMAP, probably by nerve trunk or roots activation No detection of activity in lower muscles TsMS can easily and safely stimulate phrenic nerves
**Cros 1990 [19]**	21 healthy controls	9 cm coil at C3-6	None	CMAP latencies were comparable to F waves for biceps and triceps and shorter than TES elicited. No descending tract stimulation. TsMS can stimulate motor roots exiting the spinal cord or anterior horn itself
**Britton 1990 [18]**	5 healthy controls	12 cm circular coil at C6/7, C4/5, L2 and S3	None	Reliable CMAPs from roots exiting targeted levels with shorter latencies than F waves Supramaximal responses could not be obtained. No supramaximal response was obtained
**Chokroverty 1991 [15]**	8 healthy controls and 4 patients with neck pain	9.2 cm circular coil at C7	None	TsMS latencies were consistent despite coil position and intensity, latencies were always shorter latencies than F waves. TsMS would elicit CMAP around 2.7 cm from the anterior horn at cervical level.
**Zwarts 93 [20]**	7 healthy controls	7.5 cm circular coil at low cervical column	None	Sensory potentials can be elicited by TsMS. Stimulation site appears to be near the spinal foramina Proximal retrograde sensory conduction can also be measured with TsMS
**Chokroverty 1993 [17]**	11 healthy controls, 11 patients with low back pain	9.2C cm circular coil at L5 or S1	None	TsMS would elicit CMAP around 20 cm from the spinal motor neuron at L5-S1 roots. TsMS over lumbosacral spine stimulates roots before exiting neuroforamina
**Ugawa 1994 [23]**	7 healthy controls	Double cone at inion	Slight discomfort	Descending corticospinal tract can be stimulated by magnetic stimulation at the foramen magnum as stimulation could elicit consistent tibialis anterior CMAPs
**Chokroverty 1995 [16]**	12 healthy controls and 1patient with	9.2 cm circular coil at T7-9	None	TsMS latencies were consistent despite coil position and intensity and prolonged in the diabetic patient. No supramaximal response was achieved.

	diabetes mellitus			
**Tomberg 1995 [27]**	4 healthy controls	Circular coil at C7 and up to 8 cm above single pulses	None	Multisynaptic motor activation may have been achieved either by corticospinal or propriospinal systems.<br>Abductor pollicis brevis (C8-T2) EMG size and latency compatible Spinal root activation at C7 up to 4 cm above.<br>Smaller EMG with 1.5-2.5ms longer latency when 6 or 8cm above.
**Paxton 2010 [22]**	23 healthy controls	Figure of eight coil at 100% (3.5 Tesla) over C6-7	None	Sympathetic ganglia could not be stimulated. No cardiovascular changes from intense TsMS in healthy subjects.<br>TsMS was unable to elicit any descending tract response nor alter sympathetic activity
**Ruiu 2021 [28]**	18 healthy controls	90 mm Circular coil over T2-T3.	None	Resting motor threshold was lower and peak amplitudes were higher when induced current was better aligned to a flowing out the studied spinal foramina<br>No lower limb response was elicited even at 200% resting motor threshold.<br>CMAP produced by TsMS is compatible with mixed nerve stimulation at very proximal sites.

cm: centimeter; CMAP: compound muscle action potential; EMG: needle electromyography; mm: milimiter; TES: transcranial electric stimulation; TsMS: transpinal magnetic stimulation
